# Gamma-interferon-inducible, lysosome/endosome-localized thiolreductase, GILT, has anti-retroviral activity and its expression is counteracted by HIV-1

**DOI:** 10.18632/oncotarget.12104

**Published:** 2016-09-18

**Authors:** Yoshinao Kubo, Mai Izumida, Yuka Yashima, Haruka Yoshii-Kamiyama, Yuetsu Tanaka, Kiyoshi Yasui, Hideki Hayashi, Toshifumi Matsuyama

**Affiliations:** ^1^ Division of Cytokine Signaling, Graduate School of Medical Sciences, Nagasaki University, Nagasaki, Japan; ^2^ Department of AIDS Research, Institute of Tropical Medicine, G-COE, Nagasaki University, Nagasaki, Japan; ^3^ Department of Immunology, Graduate School and Faculty of Medicine, University of the Ryukyus, Okinawa, Japan; ^4^ Present address: Department of Gastroenterology, Beth Israel Deaconess Medical Center, Harvard Medical School, Boston, MA, USA

**Keywords:** gamma-interferon, antiviral, endosome, retroviruses, thiolreductase

## Abstract

The mechanism by which type II interferon (IFN) inhibits virus replications remains to be identified. Murine leukemia virus (MLV) replication was significantly restricted by γ-IFN, but not human immunodeficiency virus type 1 (HIV-1) replication. Because MLV enters host cells via endosomes, we speculated that certain cellular factors among γ-IFN-induced, endosome-localized proteins inhibit MLV replication. We found that γ-IFN-inducible lysosomal thiolreductase (GILT) significantly restricts HIV-1 replication as well as MLV replication by its thiolreductase activity. GILT silencing enhanced replication-defective HIV-1 vector infection and virion production in γ-IFN-treated cells, although γ-IFN did not inhibit HIV-1 replication. This result showed that GILT is required for the anti-viral activity of γ-IFN. Interestingly, GILT protein level was increased by γ-IFN in uninfected cells and *env*-deleted HIV-1-infected cells, but not in full-length HIV-1-infected cells. γ-IFN-induced transcription from the γ-IFN-activation sequence was attenuated by the HIV-1 Env protein. These results suggested that the γ-IFN cannot restrict HIV-1 replication due to the inhibition of γ-IFN signaling by HIV-1 Env. Finally, we found that 4,4′-dithiodipyridine (4-PDS), which inhibits S-S bond formation at acidic pH, significantly suppresses HIV-1 vector infection and virion production, like GILT. In conclusion, this study showed that GILT functions as a host restriction factor against the retroviruses, and a GILT mimic, 4-PDS, is the leading compound for the development of novel concept of anti-viral agents.

## INTRODUCTION

When host cells detect viruses, the cells express the type I interferons (IFNs) including α- and β-IFNs. The type I IFNs induce various anti-virus host factors and protects hosts from the viruses. The mechanisms by which the type I IFNs restrict virus replications have been vigorously studied, and many host restriction factors against human immunodeficiency virus type 1 (HIV-1) have been identified already. APOBEC3G induces C to T mutations on the HIV-1 DNA genome [1]. Tetherin inhibits release of HIV-1 particles [2]. IFITM1 [3] and MX2 [4] are induced by the type I IFNs, but their restriction mechanisms against HIV-1 are not clear yet. It has been recently reported that GBP5 induced by the type I IFNs inhibits processing and virion incorporation of the HIV-1 envelope (Env) protein [5].

The type II IFN, γ-IFN, has also anti-virus activity. Although almost all cell types express the type I IFN, limited cells such as T lymphocytes and NK cells only express the type II IFN. In contrast, the type II receptor is expressed in many types of cells. The γ-IFN has critical roles in cellular immunity induced by cytotoxic T lymphocytes and macrophages. Thus, though the mechanisms of γ-IFN in cellular immunity are extensively studied, its mechanism to inhibit virus replication remains to be examined.

Previously we have reported that MLV infection occurs through endosomes like other enveloped viruses [6], showing that endosomes are important organelles for infection by many enveloped viruses. This result prompted us to speculate that several host factors among IFN-induced, endosome-localized cellular proteins restrict MLV replication. We noticed γ-IFN-inducible lysosomal thiolreductase (GILT) that is induced by γ-IFN, specifically localized to endosomes/lysosomes, and digests protein disulfide bonds at acidic pH [7–9]. Because there are many lines of evidence showing that disulfide bonds of viral Env proteins are important for their infections [10–17], we hypothesized that GILT may restrict the retrovirus replication. We found that GILT expression significantly restricts infections by replication-defective MLV and HIV vectors, and decreased transduction titers of culture supernatants from the vector-producing cells, as we expected. We report here the restriction mechanisms of GILT and the counteracting machinery of HIV-1 to this host restriction factor.

## RESULTS

### γ-IFN inhibits MLV replication but not HIV-1 replication

**Figure 1:**
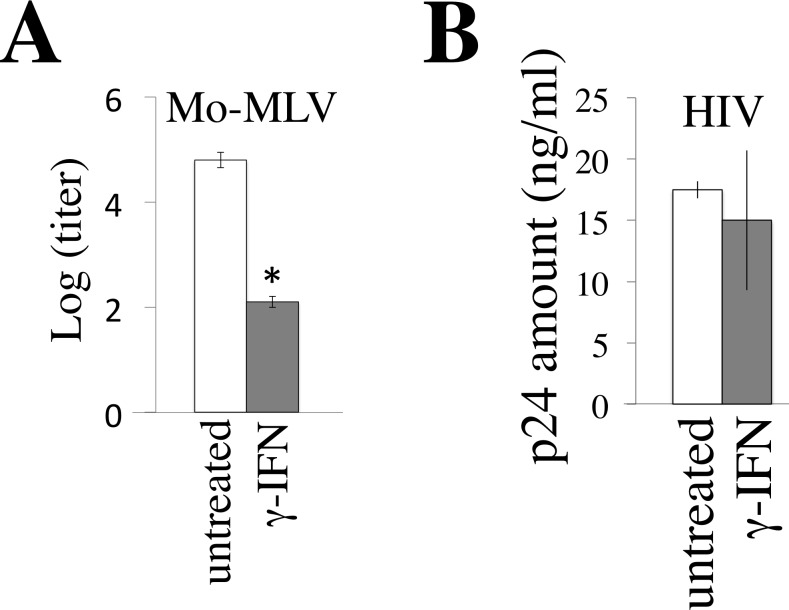
γ-IFN restricts MLV replication but not HIV-1 replication **A.** TE671/mCAT1 cells were treated with γ-IFN (0.2 μg/ml) for 1 day, and then inoculated with the Moloney MLV. The MLV titers were measured 3 days after the inoculation. **B.** TE671/CD4 cells were treated with γ-IFN (0.2 g/ml) for 1 day, and then inoculated with the HIV-1 LAI strain. The p24 amounts in culture supernatants were measured 3 days after the inoculation. These experiments were repeated three times. Means are indicated. Error bars show standard deviations. Asterisks indicate statistically significant differences.

To examine whether γ-IFN inhibits retrovirus replications, TE671 cells expressing the mouse ecotropic MLV receptor (TE671/mCAT1) and CD4 (TE671/CD4) [18] were treated with γ-IFN for 2 days, and were inoculated by the replication-competent Moloney MLV and CXCR4-tropic HIV-1 LAI strain, respectively. The treatment of TE671/mCAT1 cells with γ-IFN reduced MLV titers in culture supernatants to approximate 0.5% three days after the inoculation (Figure 1A). However, the treatment of TE671/CD4 cells with γ-IFN did not change the HIV-1 Gag p24 amounts in the culture supernatants 3 days after the inoculation (Figure 1B). These results showed that γ-IFN restricts the MLV replication, but not the HIV-1 replication.

### GILT inhibits both MLV and HIV-1 replication

**Figure 2:**
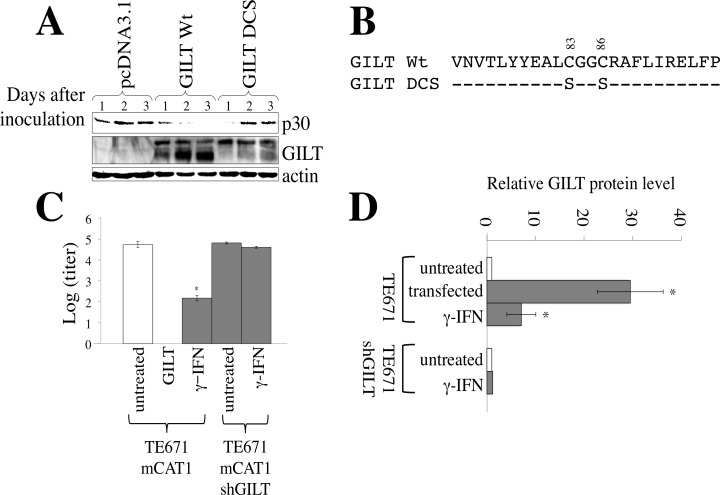
GILT inhibits MLV replication **A.** TE671/mCAT1 cells were transfected with pcDNA3.1, wild type GILT, or the DCS mutant, and inoculated with Mo-MLV. Lysates of the inoculated cells were analyzed by western blotting, using the indicated antibodies. **B.** Amino acid sequences near the thiolreductase active site are indicated. The GILT DCS mutant contains the substitutions of two cysteine residues to serine. **C.** TE671/mCAT1 cells stably transduced by the empty or shGILT-expressing lentivirus vector were treated with γ-IFN (0.2 μg/ml), and inoculated with Mo-MLV. Three days after the inoculation, Mo-MLV titers were measured by XC plaque assay (*n* = 3). Error bars show standard deviations. Asterisks indicate statistically significant differences. **D.** TE671 cells were transfected by pcDNA3.1 or GILT. TE671 cells transduced by the empty or shGILT-expressing vector were treated with γ-IFN (0.2 μg/ml). GILT and actin proteins were detected by western immunoblotting, and the intensities of the proteins were measured by a densitometer. The amounts of GILT were normalized by the actin levels. The amounts of GILT in pcDNA3.1-transfected cells are always set to 1, and relative values are indicated (*n* = 3).

As mentioned above, we speculated that γ-IFN restricts the MLV replication by inducing GILT. To assess whether GILT inhibits the MLV replication, TE671/mCAT1 cells transfected by pcDNA3.1 or GILT wild type were inoculated with the Moloney MLV, and Gag p30 levels in the cell lysates were measured by western immunoblotting. The levels of MLV Gag p30 were greatly decreased by the wild type GILT (Figure 2A). To know whether its thiolreductase activity is required for the inhibition of MLV replication by GILT, a GILT mutant (DCS) containing amino acid substitutions of two cysteine residues at the active site by serine was constructed (Figure 2B). The GILT DCS mutant did not decrease the Gag p30 amount, indicating that the thiolreductase activity is required for the GILT-induced inhibition of MLV replication. Furthermore, Moloney MLV was not detected by the XC plaque assay in culture supernatants of GILT-expressing cells 3 days after the inoculation (Figure 2C). These results showed that the GILT expression significantly suppresses the MLV replication.

In TE671 cells transfected by the GILT wild type, 40 and 30 kDa proteins bound to the anti-GILT antibody were detected. Intensities of the 40 and 30 kDa proteins were gradually decreased and increased in the GILT wild type-expressing cells, respectively. However, in the cells transfected by the GILT DCS mutant, intensities of the 30 kDa protein were much lower than those by the GILT wild type. It is known that GILT protein is synthesized as the 40 kDa precursor and then its N- and C-terminal peptides are cleaved. Thus, this result suggested that the cleavage of the GILT DCS protein is impaired, as already reported [7, 9].

To examine whether the restriction of MLV replication by γ-IFN requires GILT, the GILT expression was silenced by a lentiviral vector encoding an shRNA against the *gilt* mRNA (shGILT). The γ-IFN treatment of TE671/mCAT1 cells transduced by the empty lentiviral vector elevated GILT protein levels 7 times (Figure 2D), and significantly restricts the MLV replication. In contrast, the γ-IFN treatment of TE671/mCAT1 cells transduced by the shGILT-expressing lentiviral vector did not increase GILT protein levels, and had no effect on the MLV replication. This result showed that GILT is required for the suppression of MLV replication by γ-IFN.

**Figure 3:**
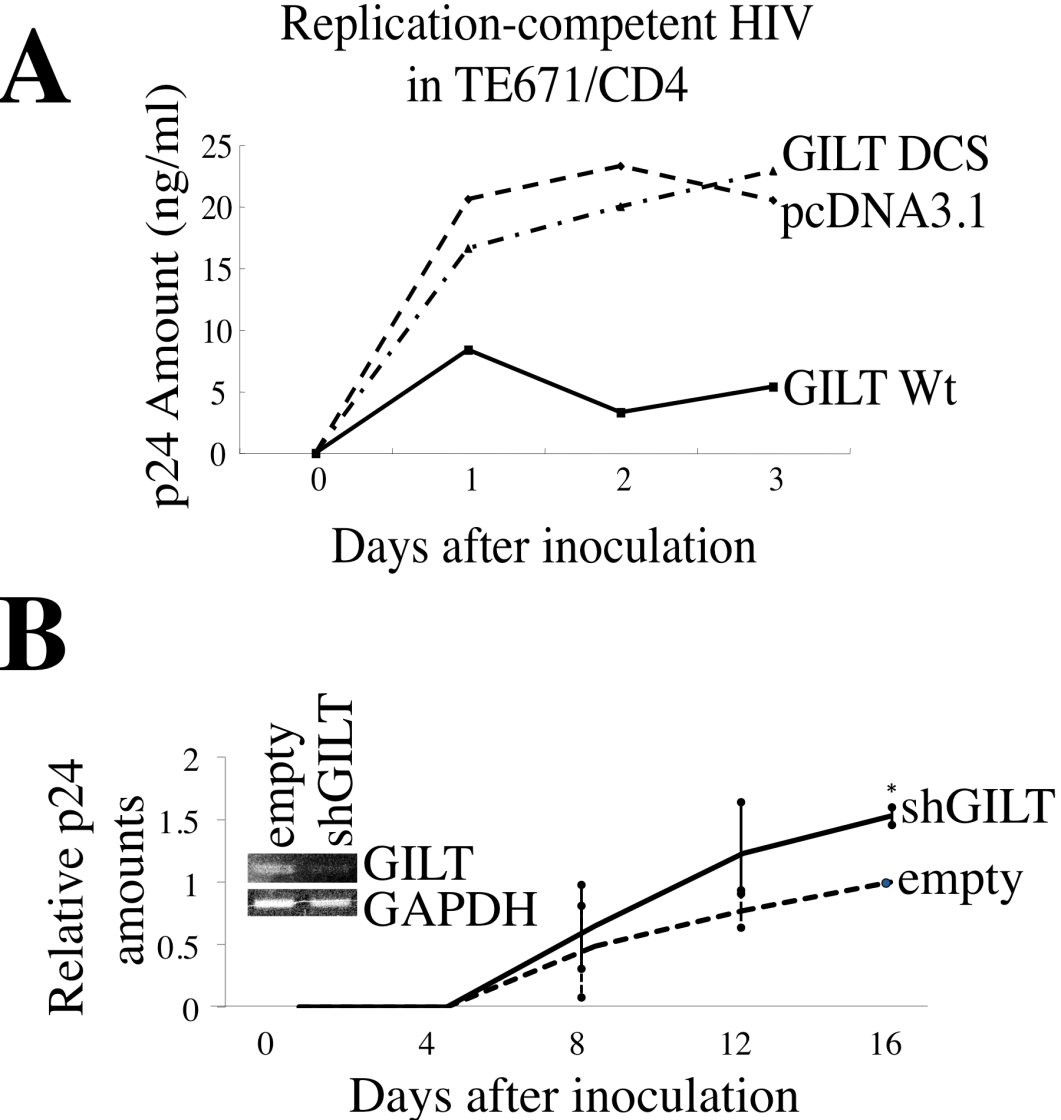
GILT restricts HIV-1 replication **A.** TE671/CD4 cells were transfected with pcDNA3.1, wild type GILT, or the GILT DCS mutant, and inoculated with the HIV-1 LAI strain. HIV-1 Gag p24 levels in the supernatants were measured. This experiment was repeated three times, and a representative result is shown. **B.** Primary MDMs transduced by the empty or shGILT-expressing lentivirus vector were inoculated with the HIV-1 AD8 strain. The amounts of Gag p24 in the supernatants were measured (*n* = 4). The amounts of p24 in the empty vector-transduced MDMs 16 days after the inoculation are always set to 1, and relative values are indicated. Asterisks indicate statistically significant differences.

To assess whether GILT inhibits HIV-1 replication, TE671/CD4 cells were transfected with the pcDNA3.1, GILT wild type, or DCS mutant expression plasmid, and then inoculated with the replication-competent HIV-1 LAI strain. The GILT expression significantly reduced the p24 levels in the culture supernatants (Figure 3A), showing that GILT restricts HIV-1 replication. In contrast, the GILT DCS mutant did not reduce the amounts of p24, indicating that the thiolreductase activity of GILT is required for the restriction of HIV-1 replication by GILT.

Macrophages constitutively express GILT. To know whether GILT expressed in macrophages restricts HIV-1 replication, primary human monocyte-derived macrophages (MDMs) were inoculated with the shGILT-expressing lentiviral vector. GILT mRNA levels in the shGILT vector-transduced MDMs were lower than those in the empty vector-transduced MDMs, analyzed by RT-PCR (Figure 3B). These cells were inoculated with the CCR5-tropic HIV-1 AD8 strain. The p24 amounts in the GILT-silenced MDMs were moderately but reproducibly higher than those in the empty vector-transduced MDMs, indicating that endogenous GILT expressed in primary human MDMs has an anti-HIV-1 activity.

### GILT inhibits viral entries by various viral envelope proteins

**Figure 4:**
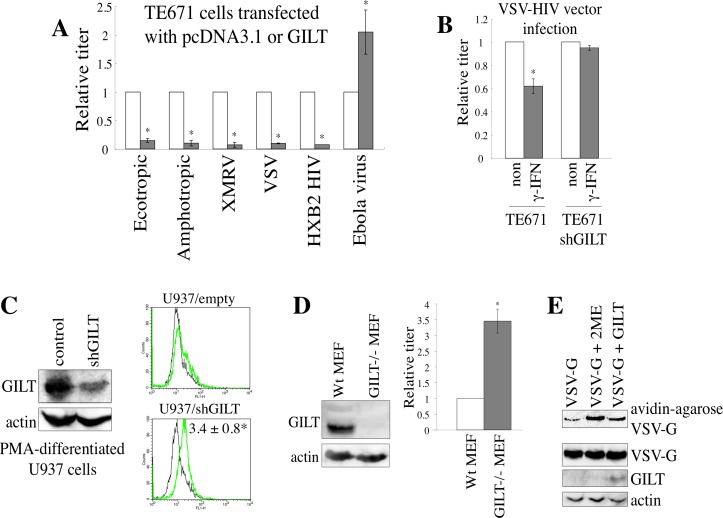
GILT inhibits viral entry by digesting S-S bonds of viral Env protein **A.** TE671 cells transfected with pcDNA3.1 (open bars) or GILT (closed bars) were inoculated with HIV-1 vector pseudotyped with indicated viral Env proteins. Relative values to titers in the pcDNA3.1-transfected cells are indicated (*n* = 3). Asterisks indicate statistically significant differences. **B.** Control and shGILT-expressing TE671 cells were treated with γ-IFN (0.2 μg/ml), and were inoculated with the VSV-pseudotyped vector. Relative values to titers in the untreated cells are indicated (*n* = 3). Asterisks indicate statistically significant differences. **C.** Control and shGILT-expressing U937 cells were treated with PMA. Lysates from the differentiated cells were analyzed by western blotting (left panel). The PMA-differentiated cells were inoculated with the VSV-pseudotyped HIV-1 vector encoding GFP, and the inoculated cells were analyzed by flow cytometry. Relative values to numbers of GFP-positive cells in control U937 macrophages are indicated (*n* = 3) (right panel). **D.** Cell lysates prepared from MEFs of GILT-knockout and wild type mice were subjected to western immunoblotting (left panel). The MEF2 were inoculated with the amphotropic MLV vector. Relative values to transduction titers in the wild type MEFs are indicated (*n* = 3). **E.** COS7 cells were transfected by VSV-G, together with pcDNA3.1 or GILT. COS7 cells transfected by VSV-G and pcDNA3.1 were treated with 2-mercaptoethanol (2ME) as a positive control. Proteins with free cysteine residues were isolated, and analyzed by western blotting (see Materials and Methods) (upper panel). Cell lysates from the transfected cells were analyzed by western blotting (three lower panels). Representative results are indicated.

Retroviral replication is a multi-step process. We next analyzed the effect of GILT on the early phase of retrovirus replication, using a pseudotyped HIV-1 vector. Infections by Env proteins of the ecotropic MLV [6], amphotropic MLV [6], xenotropic MLV (XMRV) [19], vesicular stomatitis virus (VSV) [20], and CXCR4-tropic HIV-1 HXB2 strain [21] were significantly reduced in the wild type GILT-expressing cells compared to the pcDNA3.1-transfected cells (Figures 4A and S1A), but not in the GILT DCS mutant-expressing cells (Figure S1B), showing that the thiolreductase activity of GILT expressed in the target cells confers the resistance to the infections. In contrast, when the cells were exposed to an Ebola virus-pseudotyped HIV-1 vector [22], the infection was not inhibited by GILT. These results revealed that the inhibition of pseudotyped retroviral vector infection by GILT is dependent on the viral envelope proteins, and the entry of the vector into target cells is suppressed by GILT. The cell surface expression of CD4 and CXCR4 was not changed by GILT in TE671 and 293T cells, but was rather enhanced in HeLa cells (Figure S1C), revealing that GILT inhibits the CXCR4-tropic HIV-1 Env-induced entry by a different mechanism than simply decreasing the number of receptors.

To assess whether GILT is required for the restriction of HIV-1 vector infection by γ-IFN, TE671 cells transduced by the empty or shGILT-expressing lentiviral vector were treated with γ-IFN, and inoculated with the VSV-pseudotyped HIV-1 vector. The γ-IFN treatment inhibited the VSV-G-induced infection, but the infection was not inhibited by γ-IFN treatment of the shGILT-expressing TE671 cells (Figure 4B), supporting the aforementioned results that γ-IFN restricts retrovirus replication by inducing GILT. PMA-differentiated U937 macrophages endogenously express GILT, and the GILT protein expression was largely decreased in macrophages from U937 cells stably transduced with the shGILT-expressing lentiviral vector (Figure 4C left). When a GFP-encoding VSV-pseudotyped HIV-1 vector was inoculated, the numbers of GFP-positive cells among the shGILT-expressing macrophages were higher than those among the control vector-expressing cells (Figure 4C right).

To know whether primary GILT-deficient mouse cells are more susceptible to MLV infection than wild type cells, mouse embryonic fibroblasts (MEFs) were isolated from GILT-knockout [23] and wild type mice. The GILT-deficient MEFs were more susceptible to amphotropic MLV infection than the wild type MEFs (Figure 4D). These results indicated that endogenous GILT inhibits the virus infection.

Because GILT has enzymatic activity to digest S-S bonds, it was thought that GILT inhibits the viral Env-medicated infection by digesting S-S bonds of the Env protein. To assess whether GILT indeed digests the S-S bonds of viral Env proteins, COS7 cells were transfected with VSV-G together with pcDNA3.1 or GILT, and the cells were treated with biotin-maleimide to biotinylate free cysteine residues of cell surface proteins. Biotinylated proteins were isolated using avidin-agarose, and subjected to SDS-PAGE and western blotting. As the result, the biotinylated VSV-G protein was reproducibly increased by GILT as well as 2-melcaptoethanol (2ME) (Figure 4E), indicating that GILT digests the VSV-G S-S bonds. These results suggested that GILT inhibits VSV-G-induced infection by digesting its S-S bonds.

**Figure 5:**
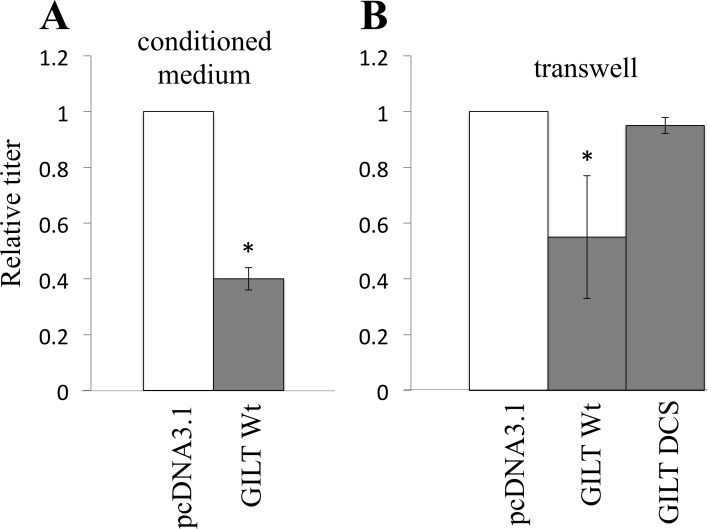
Secreted GILT inhibits viral infection **A.** VSV-pseudotyped HIV-1 vector was diluted with culture supernatant from the pcDNA3.1- or GILT-transfected COS7 cells, and inoculated to HeLa cells. **B.** Target HeLa cells were cultured with the pcDNA3.1- or GILT-transfected COS7 cells in transwells. The HeLa cells were inoculated with amphotropic MLV-pseudotyped HIV-1 vector. Relative values to titers in the pcDNA3.1-transfected cells are indicated (*n* = 3). Asterisks indicate statistically significant differences.

Secreted GILT as well as lysosome-localized GILT might inhibit the vector infection, as it has been reported that active GILT is secreted to culture media [24]. To assess the speculation, the viral vector was diluted with the culture supernatant from pcDNA3.1- or GILT-transfected COS7 cells. Transduction titers were decreased by culture supernatants from the GILT-expressing cells (Figure 5A). To confirm the result, HeLa cells were cultured with GILT Wt-, DCS-, or pcDNA3.1-transfected COS7 cells in transwells, and amphotropic MLV vector was inoculated into the HeLa cells. Transduction titers in the HeLa cells were decreased by the GILT Wt-expressing cells, but not by the GILT DCS mutant-expressing cells (Figure 5B). These results showed that secreted GILT is also involved in the restriction of vector infection.

### GILT inhibits HIV-1 virion release

**Figure 6:**
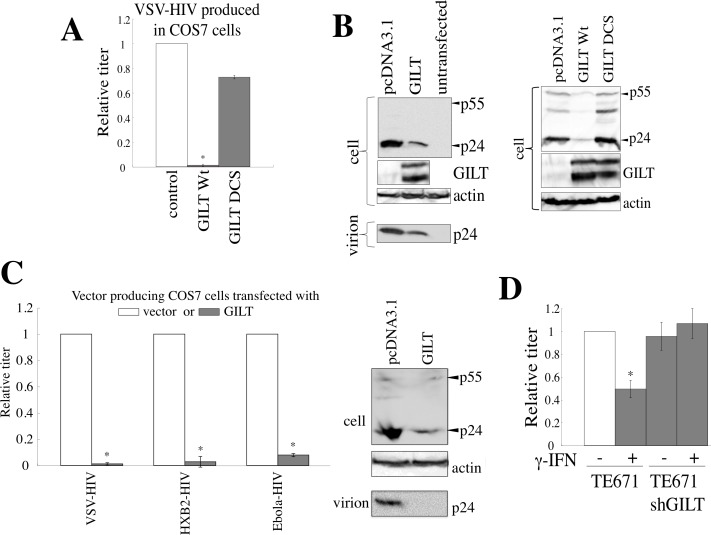
GILT inhibits HIV-1 virion production **A.** COS7 cells were transfected with the VSV-pseudotyped HIV-1 vector construction plasmids, together with pcDNA3.1, wild type GILT, or the DCS mutant. Culture supernatants from the transfected cells were used to inoculate HeLa cells, and titers were measured. Relative values to titers in the pcDNA3.1-transfected cells are indicated (*n* = 3). Asterisks indicate statistically significant differences. **B.** Cell lysates and virion pellets from the transfected cells were analyzed by western blotting. **C.** COS7 cells were transfected with the VSV-, HXB2-, or Ebola virus-pseudotyped HIV-1 vector construction plasmids together with pcDNA3.1 or wild type GILT, and transduction titers of culture supernatant from the transfected cells were measured. Relative values to titers in pcDNA3.1-transfected cells are indicated (*n* = 3). Cell lysates and virion pellets from the Ebola virus-pseudotyped HIV-1-producing cells were analyzed by western blotting. **D.** Control and shGILT-expressing TE671 cells were transfected with the VSV-pseudotyped HIV-1 vector construction plasmids, and were cultured with or without γ-IFN (0.2 μg/ml). Culture supernatants from the cells were used to inoculate HeLa cells, and the titers were measured (*n* = 3).

We then analyzed the effects of GILT on HIV-1 virion production. COS7 cells were transfected by the HIV-1 vector construction plasmids, together with the wild type GILT, DCS mutant, or pcDNA3.1. The presence of wild type GILT largely decreased both the HIV-1 titers of the culture supernatants from the transfected cells and the amounts of Gag p24 in cell lysates and culture supernatants, but the GILT DCS mutant did not (Figure 6A and 6B), showing that GILT decreases the HIV-1 Gag protein amounts and virion production. Although GILT expression in target cells did not inhibit the infection by Ebola virus glycoprotein (GP) (Figure 4A), GILT decreased transduction titers of culture supernatants from Ebola virus-pseudotyped HIV-1 vector-producing cells, as VSV- or HXB2-pseudotyped vector, and attenuated the Gag protein amount in cell lysates and culture supernatants from the transfected cells (Figure 6C). This result showed that Ebola virus GP does not neutralize the GILT function and the infection by Ebola virus GP is resistant for the anti-viral activity of GILT.

To determine whether γ-IFN inhibits HIV-1 virion production, γ-IFN was added to the TE671 cells transfected with the VSV-pseudotyped HIV-1 vector construction plasmids, for 24 hr. The cells were then washed with medium to remove the γ-IFN, and were cultured for 5 hr in the absence of γ-IFN. The titers of the culture supernatants were reduced by the γ-IFN treatment in the control cells, but not in the GILT-silenced cells (Figure 6D), revealing that γ-IFN restricts HIV-1 particle production by inducing GILT expression.

### CD63 is involved in the suppression of HIV-1 virion production by GILT

Although GILT reduced HIV-1 Gag protein amounts, GILT cannot directly bind to the Gag protein, because GILT is localized inside of endosomes/lysosomes but the Gag protein is in cytoplasm. A member of the tetraspanin superfamily, CD63, is reportedly involved in HIV-1 virion production [25–27] and is a transmembrane protein specifically localized to acidic endosomes/lysosomes [28]. The tetraspanin proteins have conserved cysteine residues in the second extracellular loop that form S-S bonds [29], and substitutions of the cysteine residues destroy their functions [30]. Therefore, GILT may inhibit HIV-1 virion production by digesting the S-S bonds in CD63.

**Figure 7:**
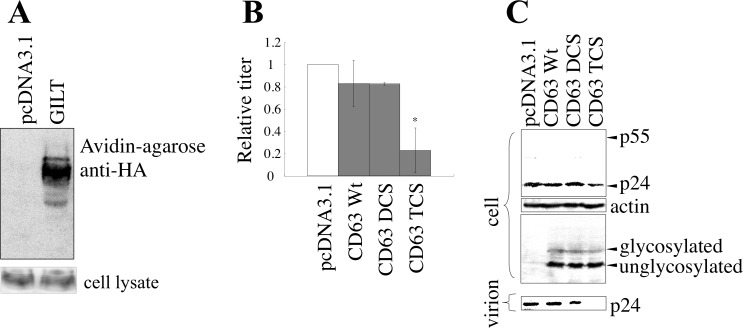
GILT inhibits HIV-1 virion production by digesting S-S bonds in CD63 **A.** COS7 cells stably expressing CD63-HA were transfected with pcDNA3.1 or GILT. Proteins with free cysteine residues were isolated, and analyzed by western blotting (upper panel). CD63 protein levels in cell lysates were also analyzed by western blotting (lower panel) **B.** COS7 cells were transfected with VSV-pseudotyped HIV-1 vector construction plasmids, together with pcDNA3.1, HA-tagged wild type CD63, or the DCS or TCS mutant, and culture supernatants from the transfected cells were used to inoculate HeLa cells. Relative values to titers in the pcDNA3.1-transfected cells are indicated (*n* = 3). Asterisks indicate statistically significant differences. **C.** Cell lysates and virion pellets from the transfected cells were analyzed by western blotting.

To determine whether GILT digests the CD63 S-S bonds, COS7 cells stably expressing C-terminally HA-tagged CD63 were transfected by pcDNA3.1 or GILT, and cell lysates prepared from the transfected cells were treated with biotin-maleimide to biotinylate proteins at free cysteine residues. The biotinylated proteins were isolated using avidin-agarose, and subjected to SDS-PAGE and western blotting. CD63 was detected exclusively in cells transfected by GILT, but not by pcDNA3.1, though GILT did not change total CD63 levels (Figure 7A). In addition, when the HA-tagged CD63 protein was analyzed by non-reducing gel electrophoresis, a larger protein band was detected (Figure S2A). Since this larger band was only detected under non-reducing conditions, this band seems to correspond to CD63 complex *via* disulfide bonds. When GILT was co-expressed, the larger protein band was not detected. These results indicated that CD63 is one of the substrates of GILT.

To assess the cellular localizations of GILT and CD63, COS7 cells were co-transfected by GILT and C-terminally GFP-tagged CD63 expression plasmids. As expected, the GILT protein co-localized with GFP-tagged CD63, as analyzed by confocal microscopy (Figure S2B). To confirm the above result, HeLa cells were transfected by the GFP-tagged CD63 expression plasmid, and treated with γ-IFN. Endogenous GILT co-localized with the GFP-tagged CD63 (Figure S2C). These results showed that GILT binds to CD63 and digests its S-S bonds.

To assess whether CD63 without disulfide bonds inhibits HIV-1 virion formation, C-terminally HA-tagged CD63 mutants containing serine substitutions of two (DCS) and four (TCS) of the six cysteine residues were constructed (Figure S3A). When COS7 cells were transfected with the VSV-pseudotyped HIV-1 vector construction plasmids together with the CD63 TCS mutant, the titers of the culture supernatants and the amounts of the Gag protein in the cell lysates and in culture supernatants were decreased (Figure 7B and 7C). However, the wild type CD63 and the DCS mutant did not change these phenomena. Analyses of the CD63 TCS mutant on reducing and non-reducing gels revealed that the CD63 TCS mutant, which inhibited the virion production, did not form the S-S bond complex (Figure S3B). However, the CD63 DCS mutant, which did not change the virion release, formed the complex in a similar manner to the wild type CD63. These results strongly supported the idea that GILT restricts HIV-1 virion release by digesting the S-S bonds in CD63.

**Figure 8:**
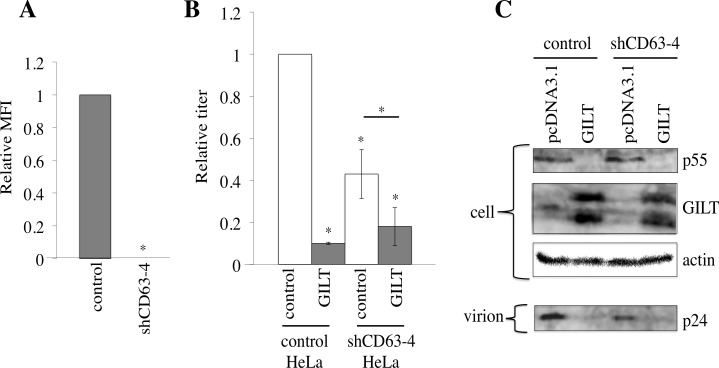
CD63 is involved in inhibition of virion formation by GILT **A.** HeLa cells were transduced by the shCD63-expressing lentiviral vector. Cell surface expression of CD63 in the shCD63- or empty vector-transduced cells was analyzed by flow cytometer. Relative values to mean of fluorescence intensities (MFIs) in the empty vector-transduced cells are indicated (*n* = 3). Asterisks indicate statistically significant differences. **B.** The empty or chCD63 vector-transduced cells were transfected with VSV-pseudotyped HIV-1 vector construction plasmids together with pcDNA3.1 or GILT. Transduction titers of the culture supernatants from the transfected cells were measured. Relative values to titers in the pcDNA3.1-transfected, empty vector-transduced cells are indicated (*n* = 3). **C.** Cell lysates and virion pellets prepared from the transfected cells were analyzed by western blotting.

To confirm the involvement of CD63 in HIV-1 virion production, endogenous CD63 expression was knocked down by the stable transduction of HeLa cells with a CD63 shRNA-expressing lentiviral vector (shCD63-4). The shCD63-4 completely abolished the cell surface expression of CD63 (Figure 8A). The titers and the Gag p24 levels in the culture supernatants from the shCD63-4-expressing cells were lower than those in the control cells, although the amounts of the Gag precursor protein in the cell lysates were similar (Figure 8B and 8C), showing that CD63 is required for efficient virion release, as reported previously [24–26]. GILT significantly (10%) reduced the transduction titers in endogenous CD63-expressing cells, but only moderately (40%) in the CD63-silenced cells. This result supported the proposal that CD63 is involved in the restriction of HIV-1 virion production by GILT.

To assess whether GILT also digest S-S bonds of CD81, another member of the tetraspanin family, COS7 cells were transfected by an expression plasmid encoding a C-terminally HA-tagged CD81 together with pcDNA3.1 or GILT. Cell lysates from the transfected cells were subjected to reducing and non-reducing SDS-PAGE followed by western immunoblotting. A protein band with higher molecular size than expected was detected in non-reducing gel but not in reducing gel, showing that CD81 forms a complex with other protein(s) through S-S bond(s) (Figure S4), like CD63. The CD81 complex was still detected in the presence of GILT. This result suggested that GILT does not digest the S-S bonds in CD81.

### GILT does not inhibit MLV particle release

**Figure 9:**
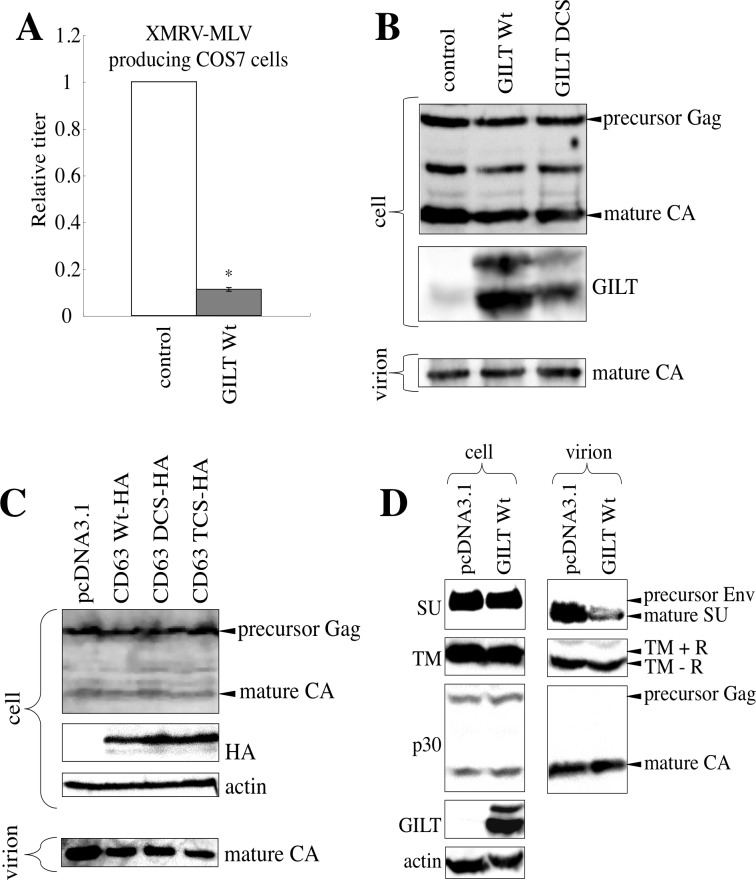
GILT decreases the infectivity of released MLV particles **A.** COS7 cells were transfected with the MLV vector construction plasmids, together with pcDNA3.1 or wild type GILT. The culture supernatants of the transfected cells were used to inoculate TE671 cells. Relative values to titers in pcDNA3.1-transfected cells are indicated (*n* = 3). Asterisks indicate statistically significant differences. **B.** COS7 cells were transfected with the MLV vector construction plasmids, together with pcDNA3.1, wild type GILT, or the DCS mutant. Cell lysates and virion pellets from the transfected cells were analyzed by western blotting, using anti-MLV p30 and anti-GILT antibodies. **C.** COS7 cells were transfected with the MLV vector construction plasmids, together with pcDNA3.1, the C-terminally HA-tagged wild type CD63, or the DCS or TCS mutant. Cell lysates and virion pellets from the transfected cells were analyzed by western blotting, using anti-MLV Gag, anti-HA, and anti-actin antibodies. **D.** COS7 cells were transfected with the MLV vector construction plasmids, together with pcDNA3.1 or wild type GILT. Cell lysates and virion pellets from the transfected cells were analyzed by western blotting, using anti-MLV p30 (CA), anti-SU, anti-TM, anti-GILT, and anti-actin antibodies. The C-terminal R peptide of the MLV TM protein is cleaved after virion budding. Therefore, the R peptide-containing TM (TM+R) and the R peptide-deficient TM (TM-R) proteins were detected in cell lysates and virion pellets, respectively.

To assess whether GILT also inhibits MLV particle release, COS7 cells were transfected by XMRV Env-containing MLV vector construction plasmids together with the pcDNA3.1, GILT wild type, or DCS mutant expression plasmid. Although transduction titers of the culture supernatants from the transfected cells were decreased by the GILT wild type (Figure 9A), the amounts of MLV Gag protein in the cell lysates and culture supernatants were not changed (Figure 9B). Consistently, the CD63 TCS mutant, which inhibited HIV-1 virion production, did not change MLV Gag protein levels in the culture supernatants (Figure 9C). To understand the mechanism by which GILT decreased transduction titers of the MLV vector without inhibiting virion production, MLV Env proteins incorporated into virions were analyzed by western immunoblotting. Interestingly, the SU subunits, but not the TM, in virion pellets was decreased by GILT (Figure 9D). Because the MLV SU subunits are anchored to virion surface by S-S bonds to the TM, this result suggested that GILT reduces infectivity of MLV virions by digesting the S-S bonds between the SU and TM proteins.

### HIV-1 Env inhibits γ-IFN signaling

**Figure 10:**
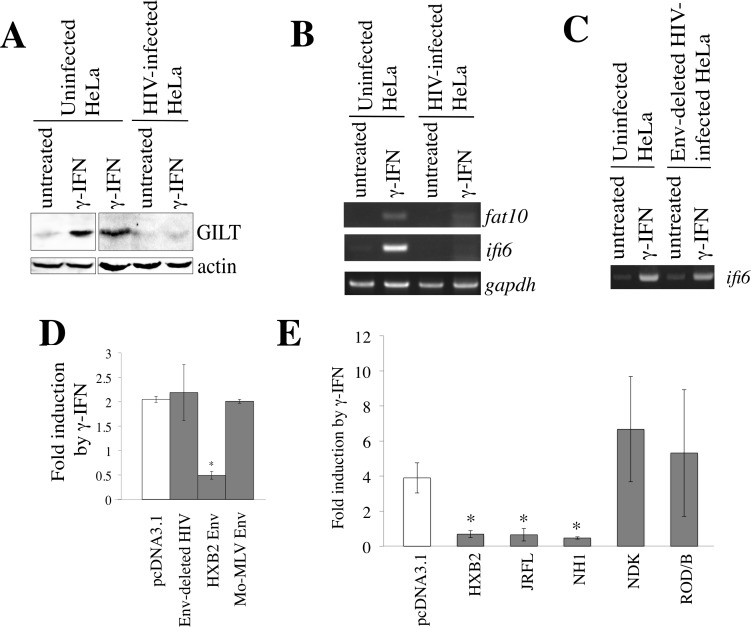
HIV-1 Env inhibits γ-IFN signaling **A.** HeLa cells were inoculated with VSV-pseudotyped replication-competent HIV-1 LAI, and then treated with γ-IFN (0.2 μg/ml). Cell lysates from the infected and uninfected HeLa cells were analyzed by western blotting. **B.** HeLa cells were inoculated with the VSV-pseudotyped HIV-1 LAI strain, and then treated with γ-IFN (0.2 μg/ml). Total RNA samples were prepared from the treated cells, and *fat10*, *ifi6*, and *gapdh* mRNA levels were measured by RT-PCR. **C.** HeLa cells were inoculated with the VSV-pseudotyped wild type or *env*-deleted HIV-1. The *ifi6* and *gapdh* mRNAs were measured by RT-PCR. **D.** and **E.** HeLa cells were transfected with CMV-R-Luc and GAS-F-Luc, together with indicated plasmids. The transfected cells were treated with γ-IFN (0.2 μg/ml), and Renilla and firefly luciferase activities were measured. The fold induction of firefly luciferase per Renilla luciferase by γ-IFN is indicated (*n* = 3). Asterisks indicate statistically significant differences.

In contrast to the fact that γ-IFN did not inhibit HIV-1 replication (Figure 1A), the GILT expression significantly restricted the vector infection by HIV-1 Env (Figure 4A) and virion production (Figure 6A). To examine whether GILT protein is induced by the treatment of HIV-1-infected cells with γ-IFN, HeLa cells were inoculated with VSV-G-pseudotyped, replication-competent HIV-1 LAI strain, and treated with γ-IFN. The γ-IFN treatment elevated GILT protein amounts in uninfected HeLa cells, but not in the HIV-1-infected cells (Figure 10A). Similarly, the γ-IFN treatment increased the mRNA levels of *fat10* and *ifi6*, other γ-IFN-inducible genes, in uninfected cells, but not in HIV-1-infected cells (Figure 10B). Because the HIV-1 infection suppressed the induction of three different γ-IFN-stimulating genes, these results suggested that HIV-1 suppresses the -IFN signaling. Interestingly, the induction of *ifi6* mRNA by γ-IFN was seen in HeLa cells infected with the VSV-pseudotyped, *env*-deleted HIV-1 mutant (Figure 10C). This result suggested that the determinant for the inhibition of γ-IFN signaling by HIV-1 is the *env* gene.

To examine whether the HIV-1 Env protein inhibits γ-IFN signaling, HeLa cells were transfected by an expression plasmid encoding the firefly luciferase under the control of the γ-IFN-activation site (GAS) of the *lpm2* gene (GAS-F-Luc) [31] and a cytomegalovirus promoter-driven Renilla luciferase expression plasmid (R-Luc), together with the indicated plasmids (Figure 10D), and then treated with γ-IFN. The ratio of firefly luciferase activity to Renilla luciferase activity was elevated by γ-IFN in the pcDNA3.1-, *env*-deleted HIV-1-, or Mo-MLV Env-transfected cells, but not in the HIV-1 Env-transfected cells. These findings clearly showed that the HIV-1 Env suppresses the γ-IFN-induced activation of GAS. This is the first demonstration that HIV-1 Env has anti-γ-IFN signaling activity.

To know which strains of HIV-1 inhibit γ-IFN signaling, an expression plasmid encoding the Env protein of the HIV-1 HXB2 (subtype B), JRFL (subtype B), NH1 (subtype AE), or NDK (subtype D) strain [21, 32] was transfected together with the GAS-F-Luc and R-Luc plasmids. An expression plasmid encoding the Env protein of the HIV-2 ROD/B strain was also used. The transfected cells were treated with γ-IFN, and firefly and Renilla luciferase activities were measured. The Env proteins of the HIV-1 HXB2, JRFL, and NH1 strains inhibited the activation of GAS transcription by γ-IFN. The Env proteins of the NDK HIV-1 and ROD/B HIV-2 strains did not inhibit it, though HIV-1 vector constructed by the transfection with the NDK or ROD/B Env expression plasmid was as infectious as that with the HXB2, JRFL, or NH1 Env expression plasmid [32]. This result suggested that the Env proteins of HIV-1 HXB2, JRFL, and NH1 strains, but not the HIV-1 NDK and HIV-2 ROD/B strains, inhibit the γ-IFN-induced GAS activation.

### Screening of 5,5′-dithiobis-2-nitrobenzoic acid-related compounds against retrovirus infection

**Figure 11:**
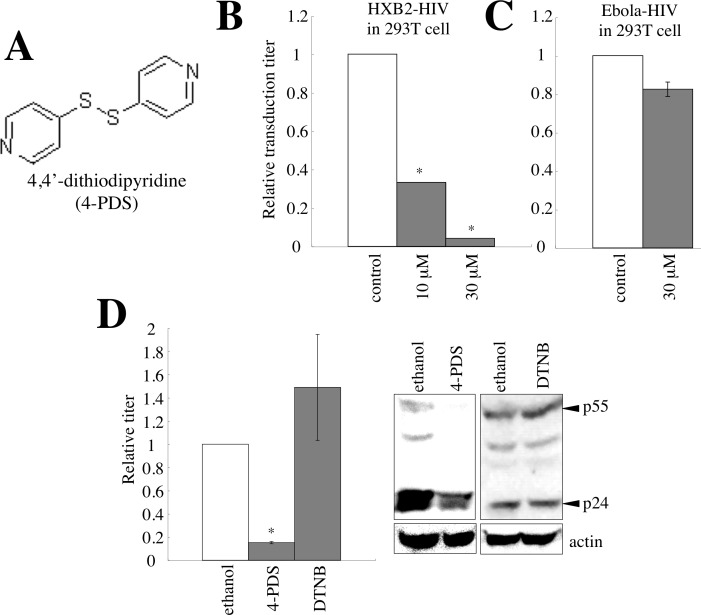
4,4′-dithiodipyridine (4-PDS) restricts the entry and virion production of HIV-1 vector **A.** Chemical structure of 4-PDS is indicated. **B.** 293T/CD4 target cells were pre-treated with 4-PDS, and inoculated with the HXB2 Env-containing HIV-1 vector. Relative values to transduction titers in solvent (ethanol)-treated cells are indicated (*n* = 3). Error bars show standard deviations. Asterisks indicate statistically significant differences. **C.** 293T cells pretreated with 4-PDS were inoculated with the Ebola virus-pseudotyped HIV-1 vector. **D.** 293T cells were transfected with the VSV-pseudotyped HIV-1 vector construction plasmids. The transfected cells were treated with 4-PDS or DTNB (30 μM) for 1 day, 24 hr after the transfection. The cells were washed with medium to remove the chemicals, and cultured for 5 hr. Transduction titers of the culture supernatants were measured. Relative values to titers in equal volume of ethanol-treated cells are indicated (*n* = 3). Cell lysates from the treated cells were analyzed by western blotting.

It has been already reported that an inhibitor of S-S bond formation, 5,5′-dithiobis-2-nitrobenzoic acid (DTNB), suppresses HIV-1 infection at mM concentrations [16]. To identify an agent that inhibits retrovirus infection more efficiently than DTNB, the amphotropic MLV vector encoding the *β-galactosidase* (β-Gal) gene as a marker was used to inoculate mouse SC-1 cells pretreated with cysteine-reacting agents (30 μM), and β-Gal activities of cell lysates prepared from the inoculated cells were measured. We found that 4,4′-dithiodipyridine (4-PDS) (Figure 11A) suppressed the infection most efficiently (Figures S5). Consistently, the infection by the HIV-1 HXB2 Env was inhibited by 4-PDS (Figure 11B). However, 4-PDS did not inhibit the infection by Ebola virus GP (Figure 11C), like GILT (Figure 4A).

GILT inhibited HIV-1 virion production. To assess whether 4-PDS also inhibits HIV-1 virion production, 293T cells transfected with the VSV-pseudotyped HIV-1 construction plasmids were treated with 4-PDS for 24 hr, 1 day after transfection. The treated cells were washed with medium to remove the 4-PDS, and were cultured for an additional 5 hr. The transduction titers of the culture supernatants were measured. The treatment of the vector-producing cells with 4-PDS, but not DTNB, reduced the titers of their culture supernatants and the amounts of Gag protein in the cell lysates (Figure 11D). These results showed that, like GILT, the 4-PDS treatment inhibits both the early and late phases of the HIV-1 life cycle.

## DISCUSSION

This study clearly showed that endogenous GILT functions as a host restriction factor against MLV and HIV-1. The restriction of MLV replication by γ-IFN required GILT. Several host defense factors have previously been identified, but they only restrict either the early or late phase of the viral life cycle. In this regard, GILT is a unique restriction factor, in that it suppresses both phases of the retrovirus replication. There are many polymorphisms within the GILT-coding region, however, it is only reported the association between rs11554159 (p.R76Q) polymorphism at the GILT locus and the risk of hyperglycemia/diabetes in severely obese individuals [33]. This is an issue requiring further study from the anti-viral point of view of GILT.

Although the GILT expression inhibited the entry and virion production of the replication-defective HIV-1 vector as efficiently as the MLV vector, γ-IFN did not restrict the HIV-1 replication. Interestingly, the HIV-1 Env protein inhibited γ-IFN signaling. This HIV-1 function can explain why γ-IFN does not suppress the HIV-1 replication. The Env proteins of the HXB2 (subtype B), JRFL (subtype B), and NH1 (subtype AE) HIV-1 strains, but not the NDK HIV-1 (subtype D) and ROD/B HIV-2 strains, inhibited γ-IFN signaling. Considering that the subtypes B and AE are circulating forms of HIV-1, it is provocative to speculate that high prevalence of HIV-1 is dependent on the strain-specific Env function to suppress γ-IFN signaling. Although there seem no specific sequence differences in the Env-coding region between the circulating and non-circulating forms, we believe that clarifying this issue is one of the keys to treatment of AIDS. It has been reported that the HIV-1 Env protein inhibits interleukin 2-induced Jak/STAT signaling [34] which is consistent with our result, but the mechanism is still unknown. Investigating the mechanism of the HIV-1 Env protein to inhibit γ-IFN is currently underway in our research group.

The γ-IFN treatment reduced vector infection 1/2 time and vector production 1/2 time. Taken together, amount of infectious viral particle released from γ-IFN-treated cells would be decreased to 1/4 of that from untreated cells in one cycle of the MLV replication. The observation that the γ-IFN treatment decreased Moloney MLV tiers to 0.5% reasonably indicated that 3 or 4 cycles of viral replication occur for 3 days. Similarly, a host restriction factor, Fv-4, slightly attenuates the MLV vector infection *in vitro*, but completely restricts the replication *in vivo* [35].

GILT inhibited the vector entry by the ecotropic, amphotropic, xenotropic, VSV, and HIV-1 Env proteins, but the entry by Ebola virus GP was rather enhanced by GILT, even though the Ebola virus GP contains disulfide bonds [36]. However, the Ebola virus GP did not alter the inhibition of HIV-1 virion production by GILT. These results suggested that the entry by Ebola virus GP is resistant to the GILT anti-virus activity, and it does not neutralize the GILT function. Further study is needed to understand the mechanism that confers GILT resistance to the entry by Ebola virus GP.

**Figure 12:**
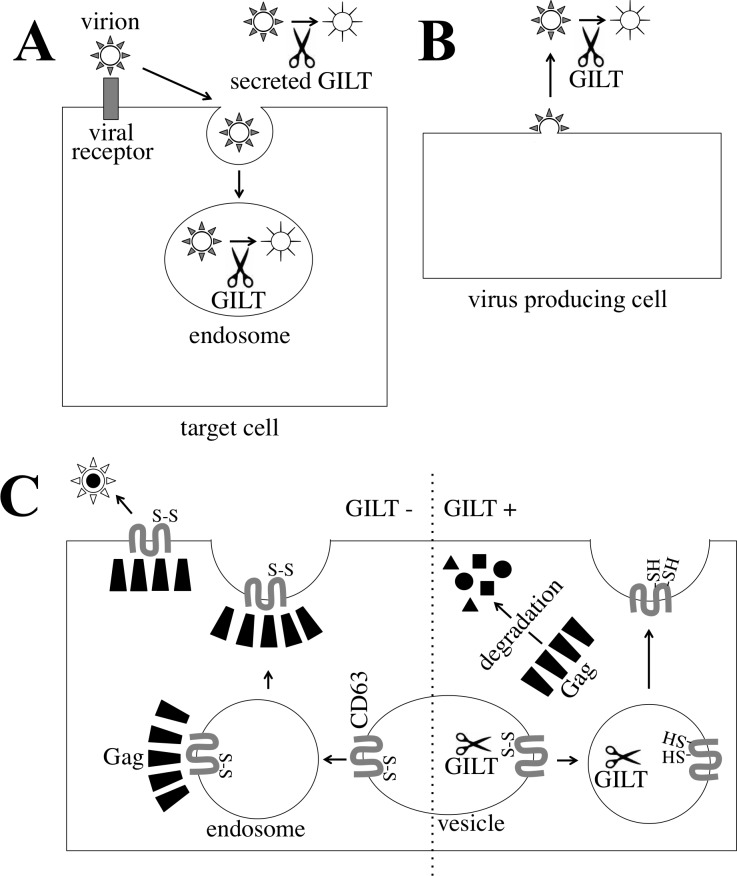
Mechanism by which GILT inhibits retrovirus replication **A.** A viral particle is internalized to an endosome of a host cell. In the endosome, GILT digests S-S bonds of the viral Env proteins, and attenuates the infection. Secreted GILT also digests S-S bonds of the viral Env proteins. **B.** GILT does not inhibit MLV virion formation, but decreases infectivity of released MLV particles. GILT digests S-S bonds of viral Env protein in the released viral particle. **C.** In GILT-negative cells, HIV-1 Gag protein forms a complex with CD63, and is transported to cell surface. Finally, virions are formed and released. In GILT-expressing cells, CD63 S-S bonds are digested, and HIV-1 Gag protein cannot form a complex with disulfide bond-deficient CD63. The free HIV-1 Gag protein is degraded by an unknown mechanism.

Taken together, this study suggested that GILT decreases the susceptibility of host cells to virus infection by digesting the Env S-S bonds of incoming virions in endosomes and culture supernatants (Figure 12A), and attenuates infectivity of released viral particles by reducing the Env disulfide bonds (Figure 12B). In addition, GILT inhibits HIV-1 virion release by digesting S-S bonds in CD63 (Figure 12C).

We also found that 4-PDS much more efficiently restricts HIV-1 entry than DTNB, but its cytotoxic effect is lower than DTNB. Furthermore, 4-PDS significantly inhibited HIV-1 virion production, but not DTNB. Because 4-PDS and DTNB are hydrophobic and hydrophilic, respectively, 4-PDS can pass through cellular membranes and reach to endosomes/lysosomes. Thus, 4-PDS may inhibit HIV-1 virion production. Because 4-PDS binds to free cysteine residues to block S-S bond formation at acidic pH, 4-PDS functions only in such acidic compartments and does not in cell surface and cytoplasm. This can explain why its cytotoxic effect was relatively lower. Taken together, our study suggested that 4-PDS is one of the leading compounds as the novel therapeutic drugs against the HIV-1-induced diseases.

It has been shown that GILT is required to digest antigen proteins to generate antigen peptides presented in MHC molecules [8, 37]. Therefore, GILT is necessary for the initiation of acquired immunity. However, GILT is conserved even in invertebrates that lack acquired immunity [38], suggesting that GILT has other functions than the initiation of acquired immunity. Together with our findings, we speculate that GILT has an evolutionarily inherent anti-viral activity.

## MATERIALS AND METHODS

### Cell lines

HeLa (human), TE671 (human), 293T (human), COS7 (human), SC-1 (mouse), and XC (rat) cells were cultured in D-MEM at 37°C in a 5 % CO_2_ atmosphere. U937 cells (human) were cultured in RPMI 1640. All of these cells were obtained from already-existing collections, and maintained in our laboratory for long time. The media were supplemented with 8 % fetal bovine serum. CD4-, mCAT1-, GILT-, or CD63-stably expressing cells were constructed with MLV vectors, as previously reported [6, 32]. To differentiate the U937 cells to macrophages, the U937 cells were treated with PMA (50 ng/ml) for 3 days.

### Primary monocyte-derived macrophages

Peripheral blood mononuclear cells (PBMCs) were prepared from a healthy donor, using LSM lymphocyte separation medium (MP Biomedicals). PBMCs were cultured in the presence of macrophage colony-stimulating factor for 7 days. Macrophages attached to the bottom of culture dishes were washed with medium, to remove floating cells.

### Screening of chemicals to inhibit virus infection

Mouse SC-1 cells were pretreated with indicated compound (Tokyo Chemical Industry) for 5 h in a 96-well plate, and were inoculated with undiluted amphotropic MLV vector encoding the β-Gal gene [6, 39]. Two days after inoculation, the β-Gal activity of the inoculated cells was measured with a highly sensitive β-galactosidase assay kit (Stratagene).

### Plasmids

The HIV-1 Gag, Pol, Tat, and Rev expression plasmid (R8.91) was kindly provided by Dr. D. Trono [40]. The β-Gal-encoding HIV-1 vector genome and VSV-G expression plasmids were kindly provided by Dr. L. Chang [20, 41]. The Ebola virus glycoprotein (GP) expression plasmid was kindly obtained from Dr. Y. Kawaoka [22]. The MLV Env expression plasmids were constructed in our laboratory [6]. The HIV-1 HXB2, JRFL, and NH1 Env expression plasmids were kindly provided by Dr. Y. Yokomaku [21]. The HIV-1 NDK Env and HIV-2 ROD/B Env expression plasmids were kindly obtained from Dr. U. Hazan [42] and Dr. P. R. Clapham [43], respectively. Human GILT and CD63 cDNA clones were isolated from HeLa cells by RT-PCR in our laboratory. Mouse GILT expression plasmids were purchased from Origene. The XMRV infectious DNA clone was provided by Dr. R. Silverman [19]. The CD81 expression plasmid was obtained from Addgene [44].

### Retrovirus replication assay

To generate replication-competent HIV-1, CD4-expressing 293T cells were transfected with an infectious molecular clone of the HIV-1 LAI strain, and were maintained until syncytia were formed. TE671/CD4 cells were then inoculated with 100 μl of the culture supernatant. To estimate the HIV-1 replication rates, the p24 levels in the culture supernatants from the inoculated cells were analyzed by ELISA (ZeptoMetrix). 293T cells were transfected with an infectious molecular clone of Mo-MLV [45], and the culture supernatant from the transfected cells was used to inoculate SC-1 cells. The titer of the culture supernatant from the infected SC-1 cells was determined by the XC test [46]. TE671/mCAT1 cells were inoculated with Mo-MLV at an MOI of 0.01. Mo-MLV replication was monitored by western blotting of lysates from the inoculated TE671/mCAT1 cells, using the anti-MLV Gag antibody (ViroMed Biosafety Laboratories).

### Retroviral vectors

The HIV-1 and MLV vectors were constructed by transient transfections of COS7 and 293T cells with the Fugene HD reagent (Promega). When the β-Gal gene was used as the reporter, the inoculated cells were stained with X-Gal (Wako) 2 days after the inoculation, to estimate the transduction titer. When the GFP gene was used as the reporter [40], the inoculated cells were analyzed by flow cytometry (BD Biosciences).

COS7 cells were transfected with the HIV-1 vector construction plasmids. The vector-producing cells were treated with recombinant human γ-IFN (0.2 μg/ml) (Wako) 24 hr after transfection, and cultured further for 24 hr. The treated cells were washed to remove γ-IFN, and cultured for 5 hr. The culture supernatants were used to inoculate the target cells, to measure the titers. Vector particles were collected by centrifugation of the culture supernatants from vector-producing cells for 5 hr through 20% sucrose.

### Target cells for vector infection

Target cells were transfected with the pcDNA3.1, wild type GILT, or GILT DCS mutant expression plasmid by the Fugene reagent (Roche). Culture supernatants from the vector-producing cells were used to inoculate the transfected cells 48 hr after transfection. The target cells were treated with recombinant human γ-IFN (0.2 μg/ml) for 48 hr, and were inoculated with the culture supernatants from the vector-producing cells.

### Detection of proteins with free cysteine residues

To detect proteins containing free cysteine residues, biotin-maleimide (Sigma-Aldrich) was used [11]. Cells transfected with VSV-G and/or GILT were treated with 2 mM biotin-maleimide, at 37°C for 30 min. The treated cells were washed with PBS to remove the biotin-maleimide, and cell lysates were prepared. Avidin-agarose (Thermo Scientific) was added to the cell lysates, and incubated at 4°C for more than 4 hr. The avidin-agarose was washed with lysis buffer, to isolate the biotinylated proteins. The biotinylated proteins were subjected to SDS-PAGE and western blotting.

Cell lysates were prepared from GILT- or pcDNA3.1-transfected COS7 cells stably expressing HA-tagged CD63. Biotin-maleimide was added to the cell lysates (final concentration 2 mM), and incubated at 37°C for 30 min. Cold acetone was then added to the cell lysates. The precipitated proteins were washed with acetone to remove the free biotin-maleimide, and solubilized in lysis buffer by ultrasonication. The biotinylated proteins were isolated by avidin-agarose.

### Reducing and non-reducing SDS-PAGE, and western immunoblotting

Cell lysates were prepared with lysis buffer containing 2 mM N-ethylmaleimide (NEM) [10]. The cell lysates were mixed with 2x sample buffer with or without 10 % 2-mercaptoethanol (2-ME) (Wako), and were subjected to SDS-PAGE (BioRad) with or without 2-ME. The fractionated proteins were transferred onto a PVDF membrane (Millipore). The membrane was treated with an appropriate primary antibody, and then with HRP-conjugated anti-mouse IgG, anti-goat IgG, or protein G (BioRad).

### Construction of GILT and CD63 mutants

The GILT and CD63 mutants were constructed by standard PCR-mediated mutagenesis (TaKaRa). The nucleotide sequences of all clones were confirmed (Applied Biosystems).

### Flow cytometry

To estimate the cell surface expression of CD4, cells were treated with an FITC-conjugated anti-CD4 antibody (Sigma-Aldrich). To analyze the cell surface expression of CXCR4 and CD63, cells were treated with rat anti-CXCR4 [47] and mouse anti-CD63 (Santa Cruz) antibodies, and then with FITC-conjugated anti-rat and Cy3-conjugated anti-mouse IgG antibodies (BioRad), respectively. The treated cells were subjected to flow cytometry (BD Biosciences).

### Cell culture in transwells

HeLa cells were cultured in a 6-well plate. Transfected COS7 cells cultured in transwells (Corstar) were added onto the HeLa cell culture. Amphotropic MLV Env-pseudotyped vector was inoculated to HeLa cells, and the cells were continued to be cultured for 2 days with the transwell culture.

### GILT knockout mice

The GILT knockout mice with C57BL/6 mouse background were kindly provided by Dr. K. Hastings [23]. The GILT knockout mice were mated with wild type C57BL/6 mice (Clear Japan), and the F1 mice were mated each other. The F2 mice expressing GILT were used as wild type mice.

### Luciferase assay

An expression plasmid encoding Renilla luciferase under the control of cytomegalovirus promoter (CMV-R-Luc) was constructed in our laboratory. A firefly luciferase expression plasmid under the control of GAS promoter (GAS-F-Luc) was kindly provided by Dr. G. R. Stark [31]. HeLa cells were transfected with the CMV-R-Luc and GAS-F-Luc vectors, together with either pcDNA3.1 or viral Env. γ-IFN was added to the transfected cells (0.2 μg/ml) 24 hr after the transfection, and the cells were cultured for 24 hr. The firefly and Renilla luciferase activities were measured using the Dual-luciferase assay kit (Promega).

### Ethics statement

PBMC was isolated from an adult volunteer. This experiment was approved by the Ethics Committee of Nagasaki University Graduate School of Biomedical Sciences (Permit Number: 11122884). The volunteer provided an informed consent that was written. The animal study was approved by the Ethics Committee of Nagasaki University (No. 0812080723) according to the Act on Welfare and Management of Animals of the government of Japan and the Regulations for the Care and Use of Laboratory Animals of Nagasaki University. All surgery was performed after natural death by Mo-MLV-induced thymoma or euthanasia by CO_2_, and we made efforts to minimize number of mice.

## SUPPLEMENTARY MATERIALS FIGURES


