# Supplementary material for: Gamma-interferon-inducible, lysosome/endosome-localized thiolreductase, GILT, has anti-retroviral activity and its expression is counteracted by HIV-1
Source: Oncotarget. 2016 Sep 18;7(44):71255–73. doi: 10.18632/oncotarget.12104 (PMC5342076; doi:10.18632/oncotarget.12104)
Supplement: Supplementary file 1 [file oncotarget-07-71255-s001.pdf]

Gamma-interferon-inducible, lysosome/endosome-localized thiolreductase, GILT, has anti-retroviral activity and its expression is counteracted by HIV-1

Supplementary Material

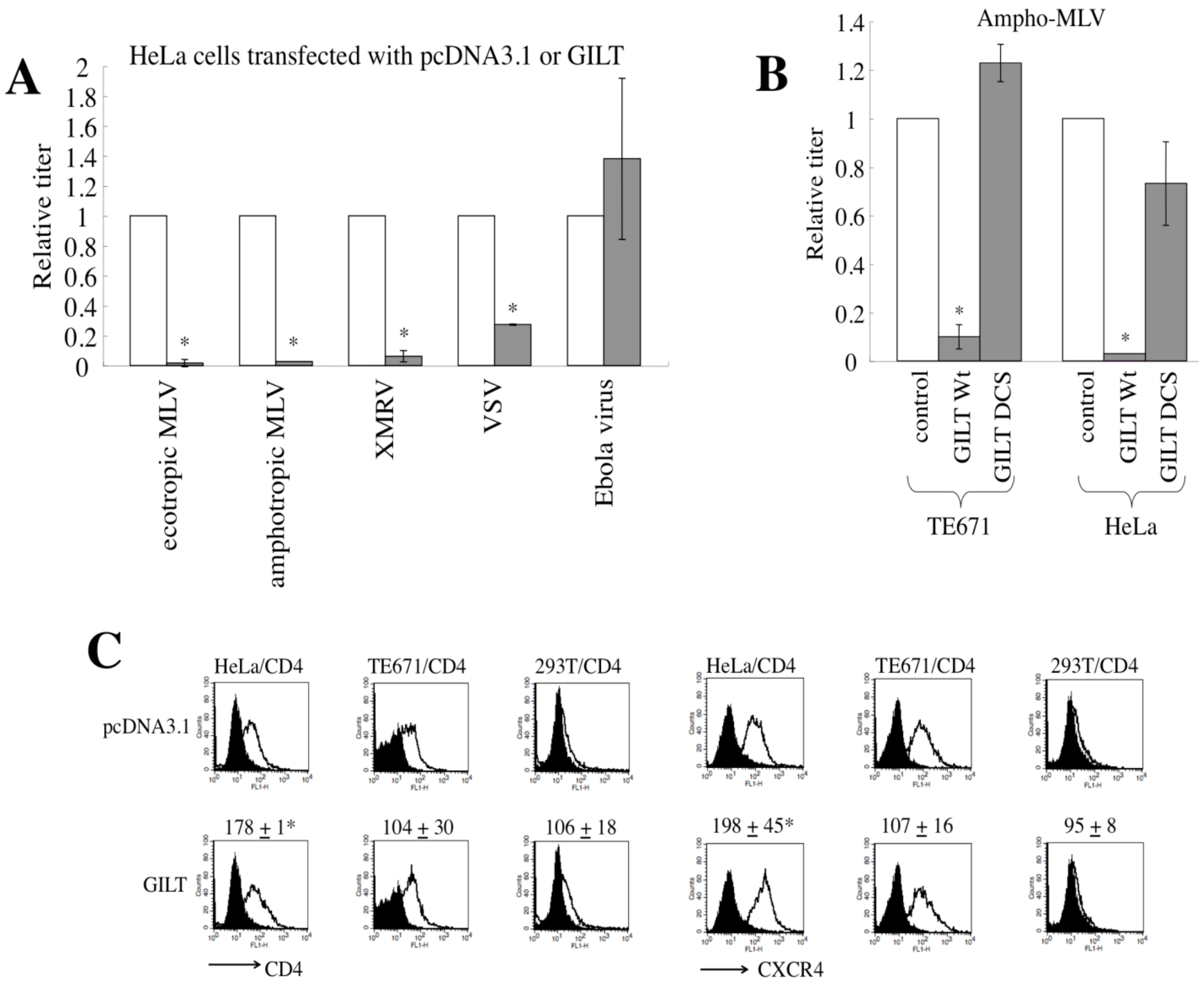

Figure S1: GILT inhibits infections by various viral envelope proteins. A. HeLa/mCAT1 cells were transfected with pcDNA3.1 or GILT, and were then inoculated with the indicated pseudotyped HIV-1 vectors. Relative values to titers in the pcDNA3.1-transfected cells are indicated (n=3). Asterisks indicate statistically significant differences. **B.** TE671 and HeLa cells were transfected by the wild type GILT, DCS mutant, or pcDNA3.1, and inoculated with an amphotropic MLV-pseudotyped HIV-1 vector. Relative values (%) to transduction titers in pcDNA3.1-transfected cells are indicated (n=3). **C.** HeLa/CD4, TE671/CD4, and 293T/CD4 cells were transfected with pcDNA3.1 or GILT. The cell surface expression of CD4 and CXCR4 was analyzed by flow cytometry. Relative values to MFI in the pcDNA3.1-transfected cells are indicated (n=3).

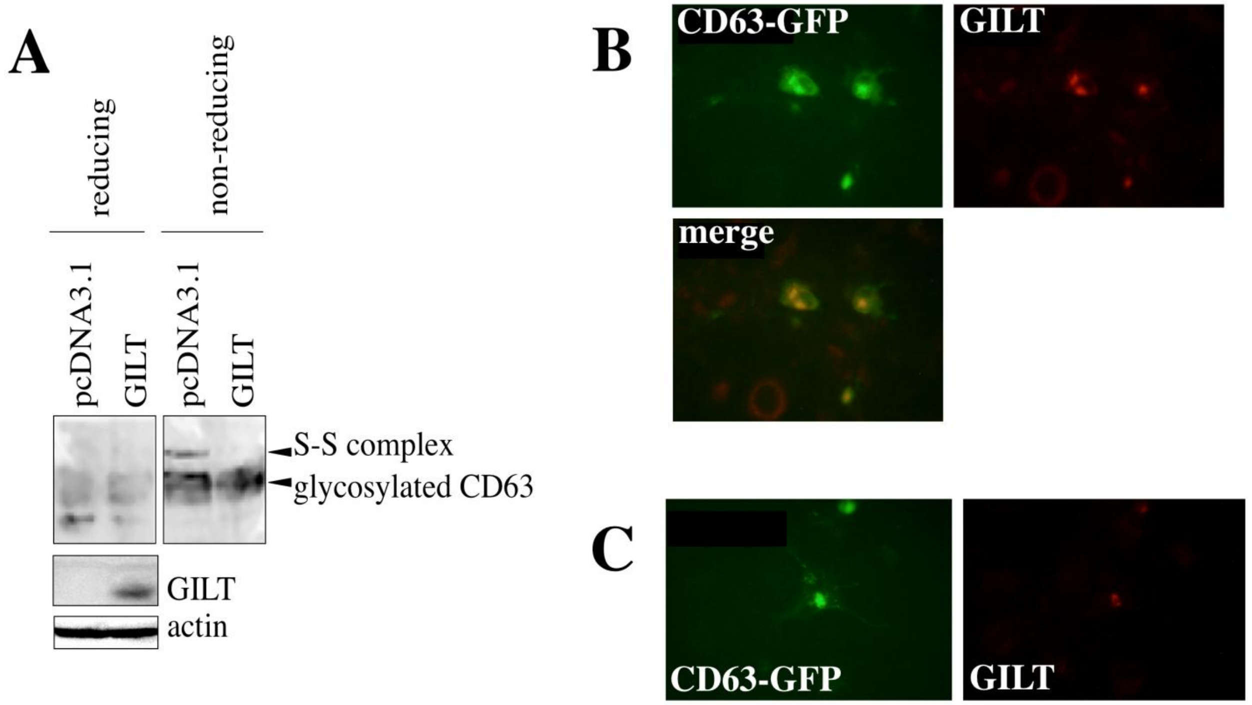

Figure S2: GILT digest CD63 S-S bonds. A. COS7 cells stably expressing HA-tagged CD63 were transfected by pcDNA3.1 or GILT. Cell lysates from the transfected cells were separated by reducing and non-reducing SDS-PAGE. HA-tagged CD63, GILT, and actin proteins were detected by western blotting. **B.** COS7 cells were co-transfected with GFP-tagged CD63 and GILT, and were immunoblotted with the anti-GILT antibody. **C.** HeLa cells transfected with GFP-tagged CD63 were treated with γ-IFN, and were immunoblotted with the anti-GILT antibody. The cells were observed by confocal microscopy.

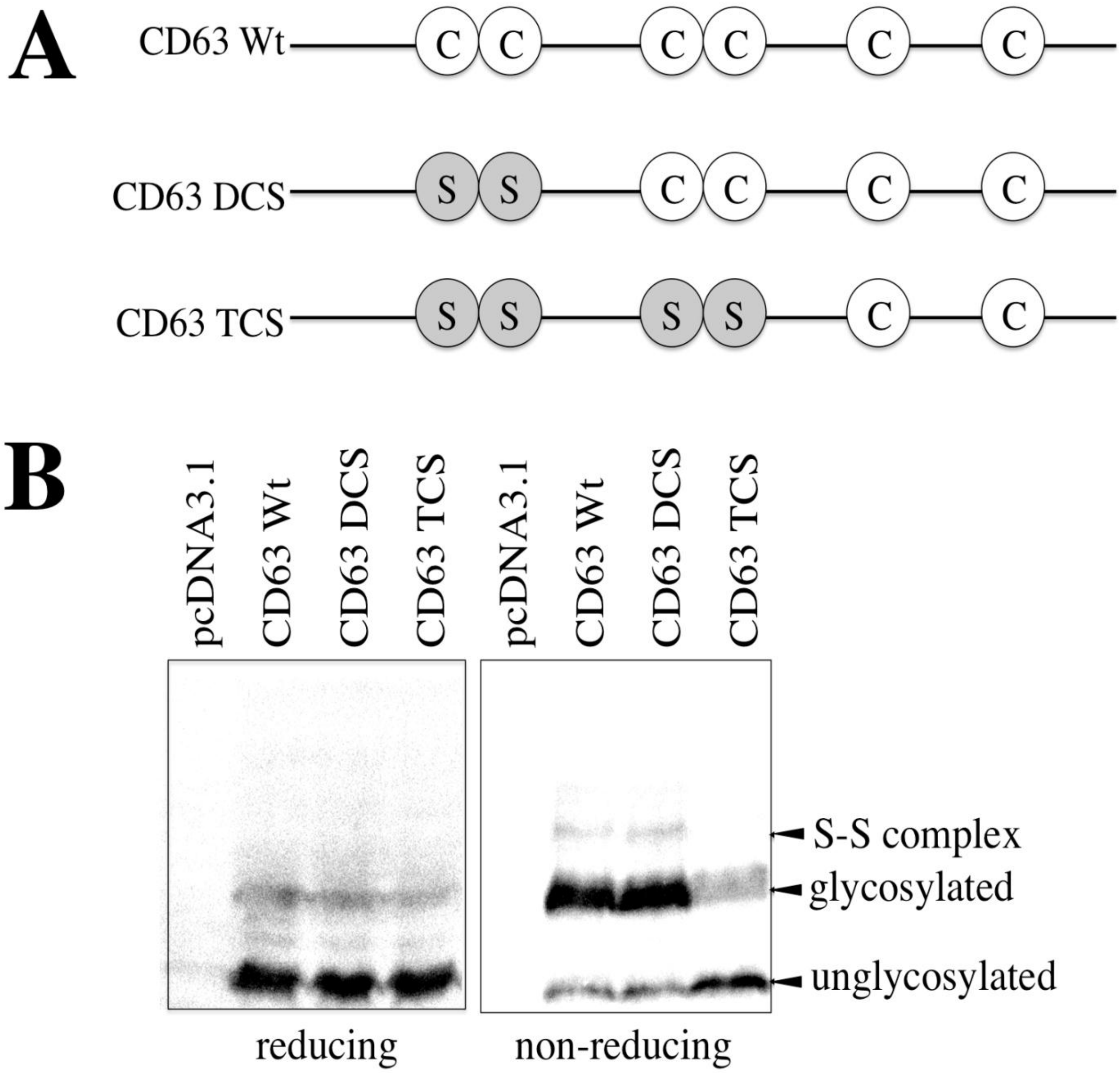

Figure S3: CD63 forms disulfide bonds. A. CD63 has six cysteine residues that form three disulfide bonds. The CD63 DCS and TCS mutants contain amino acid substitutions of the indicated two and four cysteine residues, respectively. **B.** COS7 cells were transfected with pcDNA3.1, HA-tagged wild type CD63, or the DCS or TCS mutant. The cell lysates were analyzed by reducing and non-reducing SDS-PAGE.

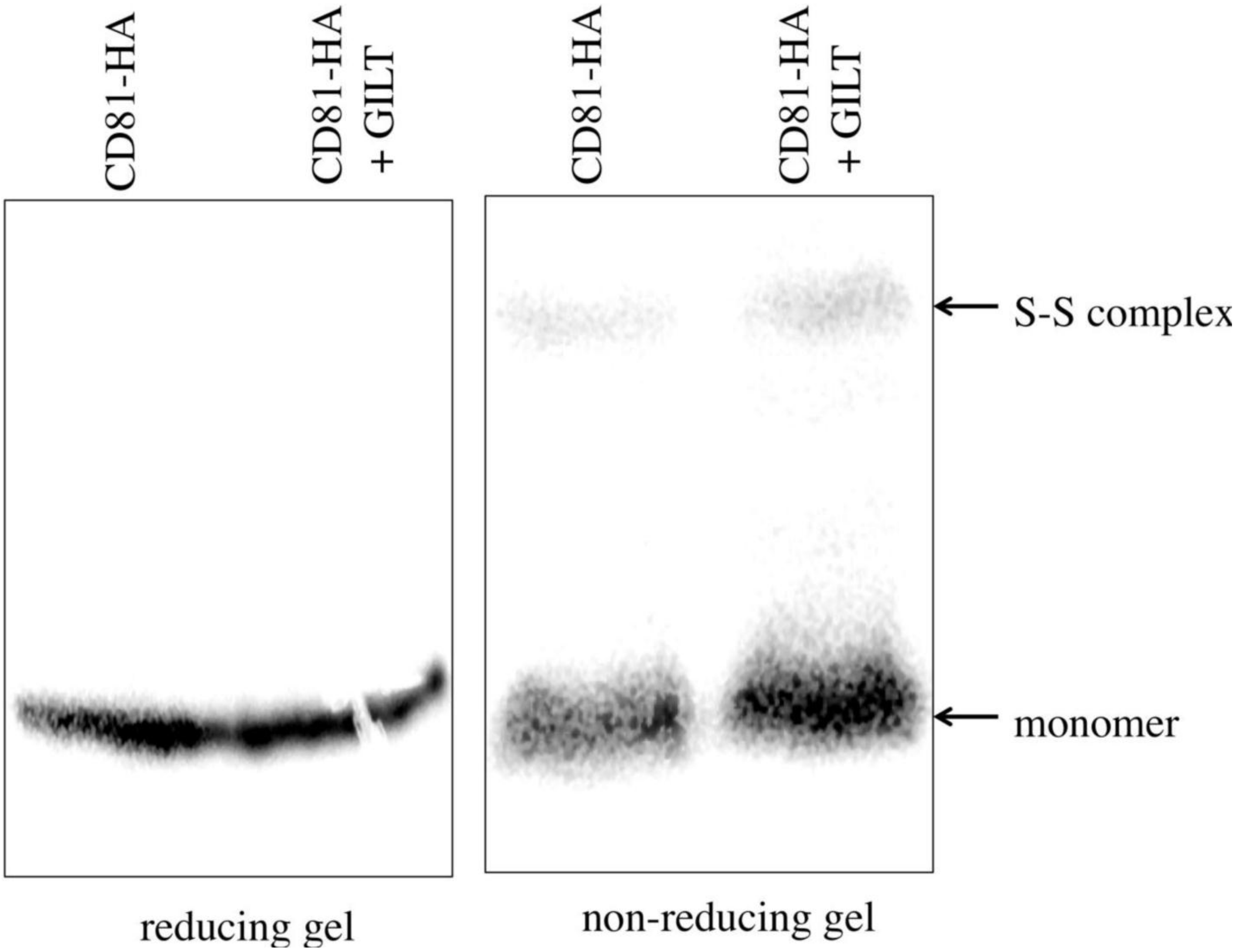

Figure S4: GILT does not digest S-S bonds in CD81. COS7 cells were transfected by C-terminally HA-tagged CD81 together with pcDNA3.1 or GILT. Cell lysates from the transfected cells were subjected to reducing (left panel) and non-reducing (right panel) SDS-PAGE followed by western immunoblotting.

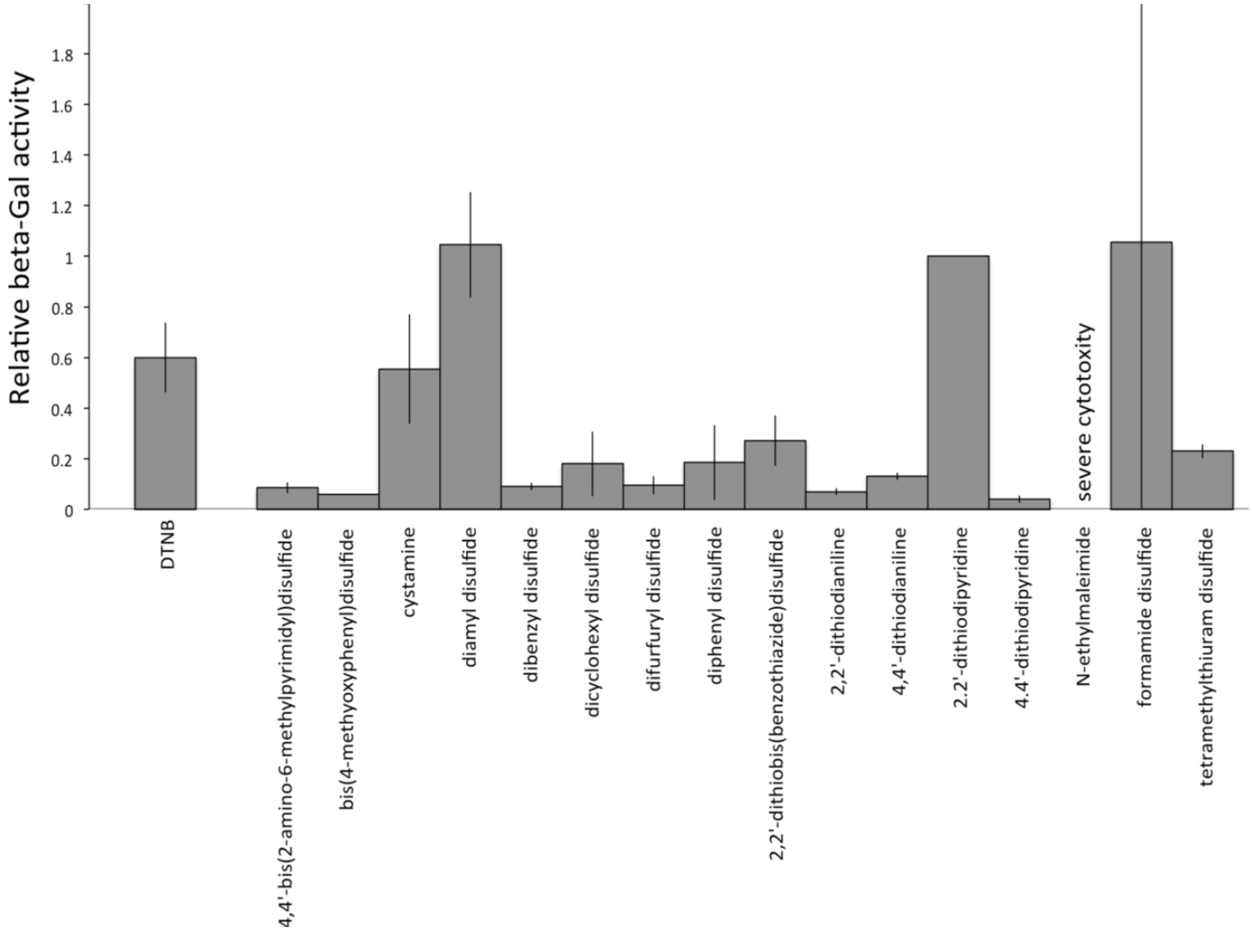

Figure S5: Screening of cysteine-reacting compounds to inhibit retrovirus infection. Mouse SC-1 cells were pretreated with the indicated compounds (30 μM) for 5 hr, and then inoculated with an amphotropic MLV vector encoding the β-*gal* gene. Relative values to the β-Gal activity in control (ethanol- or DMSO-treated) cells are indicated (n=2). Error bars indicate standard deviations.
